# Behavioral Risk Factors Associated With Overweight and Obesity Among Older Adults: the 2005 National Health Interview Survey

**Published:** 2008-12-15

**Authors:** Judy Kruger, Sandra A. Ham, Thomas R. Prohaska

**Affiliations:** Division of Nutrition and Physical Activity, Centers for Disease Control and Prevention; Centers for Disease Control and Prevention, Atlanta, Georgia; University of Illinois at Chicago, Chicago, Illinois

## Abstract

**Introduction:**

Obesity is associated with coronary heart disease, stroke, certain cancers, hypertension, and type 2 diabetes. Concern about obesity among older adults is growing, and research to examine behaviors associated with risk for increased weight in this population is needed. We examined differences by sex in behaviors associated with overweight and obesity among older adults (aged ≥50 years).

**Methods:**

We analyzed data from the 2005 National Health Interview Survey using logistic regression to predict the likelihood of overweight (body mass index [BMI], 25.0-29.9 kg/m^2^) and obesity (BMI ≥30.0 kg/m^2^) relative to healthy weight (BMI, 18.5-24.9 kg/m^2^) among older adults. We used self-reported weights and heights. Correlates were risk behaviors for chronic disease (smoking status, alcohol intake, consumption of fruits and vegetables, leisure-time physical activity, walking for leisure, walking for transportation, and strength training).

**Results:**

Among older men, the prevalence of overweight was 46.3%, and the prevalence of obesity was 25.1%. Among older women, the prevalence of overweight was 33.4%, and the prevalence of obesity was 28.8%. In adjusted logistic regression models, sex differences were observed in the significance of most risk factors for overweight and obesity. Men who were occasional, light, or moderate drinkers were 28% more likely to be obese than men who were nondrinkers; women who were heavy drinkers were 55% less likely to be obese than women who were nondrinkers. Compared with men and women who were regularly active during leisure time, inactive men were 39% more likely to be obese, and inactive women were 28% more likely to be obese.

**Conclusion:**

Several risk behaviors for chronic disease appear to be associated with overweight and obesity among older adults. Modification of these behaviors has the potential to reduce weight.

## Introduction

Between 1990 and 2000, the percentage population growth for adults aged 50 to 54 years was larger than for any other 5-year age group (1). Moreover, the prevalence of overweight and obesity among adults in every age group increased notably (2), and the prevalence of obesity among adults aged 60 years or older is expected to rise, reaching an estimated 37% in 2010 (3). Because of the growth of the aging population and a rise in the prevalence of overweight and obesity, modifying risk factors for and consequences of excess weight in older adults is critical.

Obesity is a complex, multifactorial condition that is having an increasingly negative impact on the US health care system because of the rising health care costs and lost productivity that result (4). Overweight and obesity are related to morbidity, mortality, poor quality of life, and many other problems (5) and present complex challenges for chronic disease prevention and health promotion. Relative risk for mortality among obese and overweight adults has been shown to be lower among adults aged 70 years or older than among adults aged 60 to 69 years and 25 to 59 years (6). However, obese older adults are more likely to experience functional impairment, including impairments in strength, lower body mobility, and activities of daily living than older adults at a normal weight (7), and overweight and obese older adults are more likely to become disabled than older adults at a normal weight (8). Several international studies have examined factors that may be associated with overweight and obesity among older adults (9-11). To our knowledge, only a few US studies have investigated factors associated with overweight and obesity in older adults, and these examined specific health conditions (12) or focused on psychosocial correlates (13).

We examined several behaviors (eg, inactivity, consumption of fruits and vegetables, alcohol intake) associated with overweight or obesity. Because former smokers may increase their likelihood of gaining weight when they quit smoking (former smokers have a higher body mass index [BMI] than do current smokers [14]), we included smoking status as a risk behavior. Men and women differ in their attitudes about preventive measures and health conditions (12), so we present our results stratified by sex. The aim of this study is to identify differences by sex in behavioral correlates of overweight and obesity in a large national sample of adults aged 50 years or older.

## Methods

### Sample

The National Health Interview Survey (NHIS) is an annual cross-sectional survey of noninstitutionalized civilian adults in the United States. This in-person survey of approximately 40,000 households is administered using a stratified, multistage area probability design and covers all 50 states and the District of Columbia. In 2005, the total adult response rate was 69.0% (15). We analyzed data for 13,480 adults aged 50 years or older; respondents who were underweight (BMI <18.5 kg/m^2^) or for whom data on height or weight were missing were excluded from the analysis (n = 870). Our final study sample consisted of 12,610 participants.

We included measures of demographics, BMI, functional health, and health risk behaviors. We calculated BMI from participants' self-reported heights and weights and divided respondents into 3 categories: healthy weight (BMI, 18.5-24.9 kg/m^2^), overweight (BMI, 25.0-29.9 kg/m^2^), and obese (BMI ≥30.0 kg/m^2^). Functional limitation was assessed by a single summary measure that combines people who report any difficulty with 1 or more functional activities (eg, standing for 2 hours, carrying a 10-lb object). Functional limitation was categorized as absent (not limited in any way) or present (limited in any way). Overall health status was assessed by 1 question that asked respondents about their general health status; responses were dichotomized as "good to excellent" (responses of good, very good, or excellent) or "poor to fair."

We assessed smoking status on the basis of lifetime and current cigarette use and determined 3 categories: nonsmoker (never smoked), former smoker (ex-smoker), and current smoker. We assessed alcohol intake on the basis of lifetime and current alcohol use and determined categories of "none" (no alcohol use or former drinker), "occasional, light, or moderate" intake, and "heavy" intake. We used the *Dietary Guidelines for Americans, 2005* (*Guidelines*) (16) to determine drinking levels for men and women. The *Guidelines* recommends a sex-specific cutpoint of 1 drink or fewer per day for women and 2 drinks or fewer per day for men. Respondents who consumed alcohol in excess of the recommended levels were considered heavy drinkers. For fruit and vegetable intake, respondents were asked how frequently they consumed the following foods during the past month: fruit, fruit juice, green salad, potatoes (excluding french fries, fried potatoes, and potato chips), and other vegetables. We calculated servings per day of fruits and vegetables and divided results into approximate tertiles: fewer than 2, 2 to fewer than 3.5, and 3.5 or more.

We assessed physical activity in 4 areas: 1) leisure-time physical activity, 2) walking for leisure, 3) walking for transportation, and 4) muscle-strengthening activities. Leisure-time physical activity was assessed with questions about participation, frequency (per day, week, month, or year), and duration (number of minutes, hours) at vigorous and moderate intensities. We created 3 levels, using current guidance on adequate amounts of physical activity to promote general health (engaging in ≥30 minutes of moderate-intensity physical activity on ≥5 days per week and/or ≥20 minutes of vigorous-intensity physical activity on ≥3 days per week). Participants who met this criterion were categorized as meeting the "recommended" level (17,18). We categorized participants as "insufficient" if they reported some activity but at less than the recommended level and as "inactive" if they reported no physical activity in a usual week.

Participants' walking for leisure was assessed through questions about walking outdoors for at least 10 minutes at a time for fun, relaxation, exercise, or to walk a dog (dichotomized as yes and no). Walking for transportation was assessed through questions about the number of days of walking during the previous week and the average total daily duration of trips (ie, walking to work or school, to a store or to do an errand, to the bus, or to a neighbor's house) that took at least 10 minutes. We categorized respondents as walking for transportation (yes) if they reported such walking on 5 or more days in the past week for 30 minutes or more each day and as no if they did not.

Substantial evidence exists that adults should adhere to a weight-training program that involves repetition and progressive overload on 2 or more days per week to increase muscle strength (19,20). Muscle-strengthening activities included activities such as lifting weights or doing calisthenics. Respondents were asked to report the frequency they engaged in strength training (per day, week, month, or year). Respondents were considered to have engaged in strengthening activity (yes) if they reported 2 or more days per week.

### Statistical analysis

We reported descriptive statistics dichotomized by sex for age, race/ethnicity, education level, family income, functional limitation status, and overall health status. We estimated prevalence for all of the functional health and behavioral risk-factor measures, stratified by BMI category (ie, healthy weight, overweight, and obese) and reported separately for men and women. We computed significant differences between BMI groups for each risk factor using pairwise comparisons (differences in proportions using *t* test) with α = .05. We examined correlates of overweight and obesity using logistic regression to identify the odds of being overweight or obese (using healthy weight as the referent) for each of the health risk behaviors. We assessed weight category by sex in models adjusting for demographic factors, functional health (includes both functional limitation and overall health status), smoking status, alcohol intake, servings per day of fruits and vegetables, and physical activity. Prevalence estimates and logistic regression models were weighted to account for probability of selection and nonresponse. We used SUDAAN version 9.0 (RTI International, Research Triangle Park, North Carolina) statistical software to account for the complex sampling design.

## Results

Among older men, the prevalence of overweight was 46.3%, and the prevalence of obesity was 25.1%. Among older women, the prevalence of overweight was 33.4%, and the prevalence of obesity was 28.8% (data not shown). Descriptive statistics for respondents are shown in Table 1. More women than men aged 50 years or older participated, and 39.8% of the sample was aged 50 to 59 years. Just over three-quarters of participants (75.9%) were non-Hispanic white, and approximately two-thirds (65.5%) had an annual family income of $20,000 or more. Most participants (71.2%) did not have a functional limitation, and most (77.5%) reported their health status as being good to excellent.

In adjusted analyses (Table 2), significant differences were found between obese men and men who were overweight or who were at a healthy weight. More obese men (28.8%) had a functional limitation than men who were at a healthy weight (25.6%) or men who were overweight (18.7%). More obese men (47.5%) were former smokers than were overweight men (43.9%) or men at a healthy weight (38.0%). A larger percentage of healthy-weight men (42.9%) were nondrinkers than were overweight men (36.7%) or obese men (38.0%). Differences in fruit and vegetable consumption by BMI category were also found. More obese men (36.8%) consumed fewer than 2 servings of fruits and vegetables per day than men at a healthy weight (30.0%) or men who were overweight (31.3%). Conversely, a higher percentage of healthy-weight men (30.3%) consumed 3.5 or more servings of fruits and vegetables per day than overweight men (26.4%) or obese men (19.6%). In addition, a higher percentage of healthy-weight or overweight men engaged in recommended levels of leisure-time physical activity and strength training than obese men. Correspondingly, a lower percentage of obese men (7.3%) engaged in walking for leisure than men at a healthy weight (14.6%) or men who were overweight (13.7%). Similarly, a lower percentage of obese men engaged in walking for transportation than did healthy-weight or overweight men.

As was the case with the men, many significant differences were found among women after adjusted analyses were conducted (Table 3). The percentages of women with a functional limitation increased as BMI increased (22.5% for healthy-weight women, 26.9% for overweight women, and 38.6% for obese women). More women at a healthy weight (15.3%) were current smokers than were overweight women (13.2%) or obese women (11.3%). In addition, a larger percentage of obese women (59.4%) were nondrinkers than were overweight women (52.6%) or healthy-weight women (47.9%). More women at a healthy weight (37.7%) consumed 3.5 or more servings of fruits and vegetables per day than did overweight women (32.9%) or obese women (31.0%). A larger percentage of healthy-weight women engaged in recommended levels of leisure-time physical activity (29.8%) than did overweight women (23.2%) or obese women (16.5%). Similarly, compared with overweight and obese women, a larger proportion of healthy-weight women walked for leisure, walked for transportation, and engaged in strength training.

Adjusted odds of overweight among the total sample of adults aged 50 years or older are presented in Table 4. Overall, overweight was significantly more likely among former smokers than among nonsmokers, among adults who consumed 2 to fewer than 3.5 servings of fruit and vegetables per day than among those who consumed 3.5 or more servings per day, among adults who did not walk for transportation than among those who did, and among adults who engaged in strength training than among those who did not. Overweight was less likely among current smokers than among nonsmokers and among heavy drinkers than among nondrinkers. Significant correlates of overweight varied by sex. Although being a former smoker was a correlate of overweight among men, it was not a correlate among women. Consuming 2 to fewer than 3.5 servings per day of fruits and vegetables was not a significant correlate of overweight among women, but it was among men. Overweight was significantly more likely among women who did not walk for leisure than among those who did and among women who did not engage in strengthening activities than among those who did, but this was not the case for men. Among men, not walking for transportation was a significant correlate of overweight.

Adjusted odds of obesity among the total sample of adults aged 50 years or older and for men and women are presented in Table 5. Overall, obesity was more likely among former smokers than among nonsmokers, among adults who consumed fewer than 3.5 servings of fruits and vegetables per day than among those who consumed 3.5 or more servings, among adults who were inactive or insufficiently active during their leisure time than among those who met the recommended levels of leisure-time physical activity, among adults who did not walk for leisure or transportation than among those who did, and among adults who did not engage in strengthening activities than among those who did. Obese older adults were less likely to be current smokers than nonsmokers and to be heavy drinkers than nondrinkers. Again, differences by sex were observed. Correlates for obesity among men but not women were being a former smoker; being an occasional, light, or moderate drinker; and being inactive or insufficiently active. Correlates for obesity among women but not men included heavy drinking, not walking for transportation, and not engaging in strength training. Not walking for leisure was a significant correlate of obesity among women and men.

## Discussion

Morbidities associated with overweight or obesity often are consequences of lifestyle choices (21) and environmental factors (22). We found that after adjusting for demographics, measures of functional health, and various behavioral risk factors, overweight and obesity were associated with tobacco use, alcohol intake, consumption of fruits and vegetables, and physical activity. These results suggest that overweight and obesity in older adults are complex conditions influenced by many factors. Moreover, factors that we found were associated with overweight and obesity in older adults, such as consumption of fruits and vegetables and physical activity, have been correlated with overweight and obesity among middle-aged adults (23). Given the increased interest in preventing weight gain and encouraging weight loss among older adults, a better understanding of the health risk behaviors associated with overweight and obesity, particularly among older adults who are overweight or obese, is important.

Our findings suggest that the association between cigarette smoking and BMI is complex. Older adult men who were former smokers were 29% more likely to be overweight and 43% more likely to be obese than were never smokers. Other studies that used self-reported data have also found that previous smokers have a higher BMI (24,25). In a cohort study, researchers noted that nicotine introduction was positively associated with satiety and fullness, and nicotine withdrawal was associated with hunger and increased food intake (26). Thus, the literature suggests a biological explanation for the association between cigarette smoking and weight gain or loss; nicotine withdrawal can lead to symptoms of irritability, anxiety, restlessness, depression, sleep disturbance, and increased appetite, which also could explain changes in BMI (27). However, because the prevalence of weight gain after smoking cessation has been found to occur in a small percentage of the population (28), more research into influences of weight gain after smoking cessation is needed.

The *Guidelines* explains risk factors that are related to overweight and obesity (16) and suggests that people reduce their caloric intake from alcohol (which provides few or no essential nutrients) to avoid excess caloric intake. We found that men who were occasional, light, or moderate drinkers were 28% more likely to be obese than were nondrinkers. Paradoxically, women who were heavy consumers of alcohol were 55% less likely to be obese than were nondrinkers. Epidemiologic studies of alcohol consumption and BMI have found variations by sex, with positive associations in men (29,30) and null associations in women (31,32). The relationship between consumption of alcohol and BMI is complex and may be confounded by other behaviors, such as smoking, dietary intake, and levels of physical activity. Breslow et al (33) found that people who consumed low quantities of alcohol consumed a healthful diet, consistent with the *Guidelines* (16). However, in another study by Breslow and Smothers (34), which examined the association between drinking patterns and BMI among current drinkers who had never smoked cigarettes, men and women who consumed alcohol most frequently had the lowest BMI, and men and women who consumed the least alcohol had the highest BMI. More epidemiologic studies are needed to explore the association between alcohol intake and overweight and obesity in older adults.

The *Guidelines* also proposes calorie-lowering strategies that include eating foods low (per weight or volume) in calories and high in fiber content, which are characteristic of many types of fruits and vegetables (16). Moreover, fruits and vegetables contain many beneficial vitamins, minerals, and phytochemicals, which are thought to protect against several chronic diseases, such as cardiovascular disease (35) and diabetes (36). We found that men who consumed 2 to fewer than 3.5 servings of fruits and vegetables per day were 27% more likely to be overweight than men who consumed 3.5 or more servings per day. Because energy intake affects weight management, encouraging older adults to increase the consumption of fruits and vegetables that have a high water content and can increase satiety may be important (37). Epidemiologic data suggest that high intake of fruits and vegetables protects against type 2 diabetes (38), and improvements in consumption of fruits and vegetables may help men and women maintain or even lower their body weight.

Regular physical activity increases muscular strength and endurance and improves gait and balance at all ages, and for both sexes (17,18). A Swedish study found that active older adults performed better functionally and experienced fewer fractures due to falls than did inactive older adults who were age-matched (39). Our findings indicate variability in the types of physical activity older men and women engage in; however, no conclusions can be drawn from these findings. Regular physical activity can help manage body weight and prevent weight gain (4), and longitudinal research suggests that adults who become overweight are likely to maintain a higher weight during their lifetime and to report less leisure-time or sports activity than those who were never overweight (40). Because research consistently shows that moderate-intensity aerobic activities (eg, walking) provide health benefits and count toward energy balance, all adults, independent of body weight, should be encouraged to engage in at least minimal levels of recommended activity (20).

The relationship between BMI and disability is complex. Compared with older adults at a healthy weight, older adults who are overweight or obese are more likely to develop various functional impairments, including diminished strength and greater difficulty moving their lower bodies (7). In addition, health problems and disability are associated with a decline in physical activity, which can lead to increased BMI. Our measures of functional health (functional limitations and overall health status) may be indicators of multiple influences on weight gain and obesity among older adults. We found that a larger proportion of obese men and women experienced functional limitations or self-reported poor to fair health compared with healthy-weight or overweight adults. Other studies found that, among older adults, the association between self-reported functional limitation increased with BMI (7,41).

Our findings are subject to several limitations. First, NHIS data are cross-sectional and do not allow any statistical relationships to be interpreted as causal. Although modifications in risk behaviors could plausibly lead to a shift in the prevalence of overweight and obesity, research has not determined with certainty the order of influence between risk behavior and weight status. Second, these data are self-reported; thus, misclassification bias may have occurred because people who are overweight and obese tend to underestimate their weight and overestimate their height (42). How this potential bias affects the estimates is unknown; however, we would expect the same general pattern to prevail (eg, overstated height, understated weight, exaggerated consumption of fruits and vegetables, overstated participation in physical activity) because of social desirability. Third, we were not able to examine all of the risk factors that may be associated with increased risk of overweight and obesity; future studies should consider other types of risk factors that are likely to affect older adults more frequently than younger people. Two examples include clusters of medical conditions and medical events (eg, diabetes complications, arthritis, and depression) and measures of social context (eg, marital/widow status). Fourth, fruit and vegetable consumption and physical activity behavior questions from NHIS have not been validated or tested for reliability.

### Conclusion

The high prevalence of overweight and obesity among the growing older adult population in the United States underscores the importance of initiatives in risk reduction and health promotion. Modification of several common behaviors, such as an increased consumption of fruits and vegetables and regular physical activity, may help reduce the risk of overweight and obesity. Our findings suggest that lifestyle changes to reduce behavioral risk factors for overweight and obesity among older adults should be promoted. Our knowledge of the effects of specific behavioral risk factors on the prevalence of overweight and obesity in the older adult population can be improved through continued public health surveillance efforts, and prevalence could well be reduced considerably by effective lifestyle modification programs that target risk factors at the population level. Population-based studies are needed to further enhance our understanding of the behaviors that are potentially useful for reducing overweight and obesity. A better understanding of barriers to reducing health risk behaviors and increasing health-promoting behaviors in the older adult population is needed, especially for those who are currently overweight or obese.

## Figures and Tables

**Table 1:** Characteristics of Adults Aged ≥50 Years (N = 13,480), National Health Interview Survey, 2005

**Characteristic**	Men, No. (%)^a^ (n = 5,711)	Women, No. (%)^a^ (n = 7,769)
**Age, y**		
50-59	2,422 (42.4)	2,942 (37.9)
60-69	1,616 (28.3)	2,054 (26.4)
≥70	1,673 (29.3)	2,773 (35.7)
**Race/ethnicity**		
Non-Hispanic white	4,218 (76.3)	5,689 (75.5)
Non-Hispanic black	709 (12.8)	1,007 (13.4)
Hispanic	600 (10.9)	839 (11.1)
**Education level**		
<High school graduate	1,142 (20.3)	1,711 (22.3)
High school graduate	1,612 (28.6)	2,497 (32.6)
Some college	1,322 (23.5)	1,909 (24.9)
College graduate	1,553 (27.6)	1,545 (20.2)
**Annual family income, $**		
<20,000	1,238 (21.7)	2,399 (30.9)
≥20,000	4,091 (71.6)	4,732 (60.9)
**Functional limitation^b^**		
Absent	4,223 (74.0)	5,358 (69.1)
Present	1,483 (26.0)	2,401 (30.9)
**Overall health status**		
Good to excellent	4,470 (78.3)	5,967 (76.8)
Poor to fair	1,236 (21.7)	1,801 (23.2)

a Percentages are unweighted. Numbers may not total to 100% because of missing data.

b People who report any difficulty with 1 or more functional activities.

**Table 2:** Prevalence of Risk Factors for Chronic Disease Among Men Aged ≥50 Years by Body Mass Index, National Health Interview Survey, 2005

**Characteristic**	Participants at a Healthy Weight^a^ (n = 1,569), Weighted % (95% CI)	Overweight^b^ Participants (n = 2,549), Weighted % (95% CI)	Obese^c^ Participants (n = 1,380), Weighted % (95% CI)
**Functional limitation^d^**			
Absent	74.4 (71.8-76.8)	81.3 (79.5-82.9)	71.2 (68.5-73.8)
Present	25.6 (23.2-28.2)	18.7 (17.1-20.5)	28.8 (26.2-31.5)
**Overall health status**			
Good to excellent	78.0 (75.6-80.2)	84.9 (83.4-86.3)	75.1 (72.5-77.5)
Poor to fair	22.0 (19.8-24.4)	15.1 (13.7-16.6)	24.9 (22.5-27.5)
**Smoking status**			
Nonsmoker	40.0 (37.3-42.8)	39.9 (37.7-42.1)	36.8 (33.9-39.8)
Former smoker	38.0 (35.3-40.9)	43.9 (41.6-46.2)	47.5 (44.5-50.5)
Current smoker	22.0 (19.6-24.6)	16.2 (14.6-18.1)	15.7 (13.5-18.2)
**Alcohol intake^e^**			
None	42.9 (39.9-46.0)	36.7 (34.4-38.9)	38.0 (35.1-41.0)
Occasional, light, or moderate	51.5 (48.5-54.4)	58.9 (56.7-61.1)	57.9 (55.0-60.7)
Heavy	5.6 (4.4-7.3)	4.5 (3.6-5.5)	4.1 (3.2-5.3)
**Servings per day of fruits and vegetables^f^**			
<2	30.0 (27.4-32.8)	31.3 (29.3-33.4)	36.8 (33.6-40.1)
2 to <3.5	39.7 (36.7-42.7)	42.3 (40.0-44.6)	43.7 (40.5-46.9)
≥3.5	30.3 (27.4-33.3)	26.4 (24.4-28.5)	19.6 (17.0-22.4)
**Leisure-time physical activity^g^**			
Inactive	44.9 (41.9-48.0)	40.6 (38.4-43.0)	48.6 (45.3-52.0)
Insufficient	24.5 (22.1-27.0)	28.4 (26.4-30.5)	31.7 (28.9-34.6)
Recommended	30.6 (27.9-33.5)	31.0 (28.8-33.2)	19.7 (17.1-22.5)
**Walking for leisure^h^**			
Yes	14.6 (12.6-16.8)	13.7 (12.3-15.3)	7.3 (5.8-9.2)
No	85.4 (83.2-87.4)	86.3 (84.7-87.7)	92.7 (90.8-94.2)
**Walking for transportation^i^**			
Yes	6.9 (5.7-8.3)	5.6 (4.7-6.8)	4.0 (2.9-5.5)
No	93.1 (91.7-94.3)	94.4 (93.2-95.3)	96.0 (94.5-97.1)
**Strength training^j^**			
Yes	16.7 (14.7-19.0)	17.2 (15.6-19.0)	11.4 (9.6-13.5)
No	83.3 (81.0-85.3)	82.8 (81.0-84.4)	88.6 (86.5-90.4)

Abbreviation: CI, confidence interval.

a Body mass index (BMI), 18.5-24.9 kg/m^2^.

b BMI, 25.0-29.9 kg/m^2^.

c BMI, ≥30.0 kg/m^2^.

d People who report any difficulty with 1 or more functional activities.

e Drinking levels were determined using the *Dietary Guidelines for Americans, 2005* (16), which recommends a sex-specific cutpoint of 1 drink or fewer per day for women and 2 drinks or fewer per day for men. Respondents who consumed alcohol in excess of the recommended levels were considered heavy drinkers.

f Respondents were asked how frequently they consumed the following foods during the past month: fruit, fruit juice, green salad, potatoes (excluding french fries, fried potatoes, and potato chips), and other vegetables. We calculated servings per day of fruits and vegetables and divided results into approximate tertiles.

g Respondents who reported engaging in ≥30 minutes of moderate-intensity physical activity on ≥5 days per week and/or ≥20 minutes of vigorous-intensity physical activity on ≥3 days per week were categorized as meeting the recommended level (17,18). Respondents who reported some activity but at a lower-than-recommended level were categorized as "insufficient," and respondents who reported no physical activity in a usual week were categorized as "inactive."

h Participants were asked whether they walked outdoors for at least 10 minutes at a time for fun, relaxation, or exercise, or to walk a dog.

i Walking for transportation was assessed with questions about respondents' number of days of walking during the previous week and the average total daily duration of trips (ie, walking to work or school, to a store or to do an errand, to the bus, or to a neighbor's house) that took at least 10 minutes. Respondents were categorized as walking for transportation (yes) if they reported such walking on ≥5 days in the past week for ≥30 minutes or more each day and as no if they did not.

j Respondents were considered to have engaged in muscle-strengthening activity (eg, lifting weights, calisthenics) (yes) if they reported 2 or more days per week.

**Table 3:** Prevalence of Risk Factors for Chronic Disease Among Women Aged ≥50 Years by Body Mass Index, National Health Interview Survey, 2005

**Characteristic**	**Participants at a Healthy Weight^a^ (n = 2,682), Weighted % (95% CI)**	**Overweight Participants (n = 2,379),^b^ Weighted % (95% CI)**	**Obese Participants (n = 2,051),^c^ Weighted % (95% CI)**
**Functional limitation^d^**			
Absent	77.5 (75.7-79.3)	73.1 (71.0-75.1)	61.4 (59.0-63.8)
Present	22.5 (20.7-24.3)	26.9 (24.9-29.0)	38.6 (36.2-41.0)
**Overall health status**			
Good to excellent	84.5 (82.9-86.0)	80.1 (78.3-81.8)	68.8 (66.4-71.1)
Poor to fair	15.5 (14.0-17.1)	19.9 (18.2-21.7)	31.2 (28.9-33.6)
**Smoking status**			
Nonsmoker	59.8 (57.8-61.8)	59.9 (57.6-62.2)	61.8 (59.5-64.1)
Former smoker	24.9 (23.2-26.8)	26.9 (24.9-29.0)	26.9 (24.7-29.2)
Current smoker	15.3 (13.8-16.9)	13.2 (11.6-14.9)	11.3 (9.8-13.0)
**Alcohol intake^e^**			
None	47.9 (45.6-50.3)	52.6 (50.3-54.8)	59.4 (56.9-61.8)
Occasional, light, or moderate	47.3 (45.0-49.5)	43.9 (41.7-46.1)	38.7 (36.3-41.2)
Heavy	4.8 (3.9-5.9)	3.6 (2.9-4.5)	1.9 (1.3-2.7)
**Servings per day of fruits and vegetables^f^**			
<2	23.2 (21.3-25.1)	25.7 (23.6-28.0)	29.5 (27.1-32.0)
2 to <3.5	39.1 (36.9-41.4)	41.4 (39.1-43.7)	39.5 (36.9-42.1)
≥3.5	37.7 (35.5-40.0)	32.9 (30.5-35.3)	31.0 (28.6-33.6)
**Leisure-time physical activity^g^**			
Inactive	42.9 (40.6-45.3)	47.4 (44.8-50.1)	55.9 (53.2-58.5)
Insufficient	27.3 (25.3-29.4)	29.4 (27.3-31.7)	27.6 (25.4-29.9)
Recommended	29.8 (27.7-31.9)	23.2 (21.1-25.3)	16.5 (14.7-18.6)
**Walking for leisure^h^**			
Yes	14.8 (13.2-16.7)	10.2 (8.8-11.8)	5.6 (4.6-6.9)
No	85.2 (83.3-86.8)	89.8 (88.2-91.2)	94.4 (93.1-95.4)
**Walking for transportation^i^**			
Yes	5.6 (4.6-6.7)	3.7 (2.8-4.7)	2.7 (2.0-3.7)
No	94.4 (93.3-95.4)	96.3 (95.3-97.2)	97.3 (96.3-98.2)
**Strength training^j^**			
Yes	18.3 (16.6-20.1)	13.1 (11.5-14.9)	11.0 (9.5-12.7)
No	81.7 (79.9-83.4)	86.9 (85.1-88.5)	89.0 (87.3-90.5)

Abbreviation: CI, confidence interval.

a Body mass index (BMI), 18.5-24.9 kg/m^2^.

b BMI, 25.0-29.9 kg/m^2^.

c BMI, ≥30.0 kg/m^2^.

d People who report any difficulty with 1 or more functional activities.

e Drinking levels were determined using the *Dietary Guidelines for Americans, 2005* (16), which recommends a sex-specific cutpoint of 1 drink or fewer per day for women and 2 drinks or fewer per day for men. Respondents who consumed alcohol in excess of the recommended levels were considered heavy drinkers.

f Respondents were asked how frequently they consumed the following foods during the past month: fruit, fruit juice, green salad, potatoes (excluding french fries, fried potatoes, and potato chips), and other vegetables. We calculated servings per day of fruits and vegetables and divided results into approximate tertiles.

g Respondents who reported engaging in ≥30 minutes of moderate-intensity physical activity on ≥5 days per week and/or ≥20 minutes of vigorous-intensity physical activity on ≥3 days per week were categorized as meeting the recommended level (17,18). Respondents who reported some activity but at a lower-than-recommended level were categorized as "insufficient," and respondents who reported no physical activity in a usual week were categorized as "inactive."

h Participants were asked whether they walked outdoors for at least 10 minutes at a time for fun, relaxation, or exercise, or to walk a dog.

i Walking for transportation was assessed with questions about respondents' number of days of walking during the previous week and the average total daily duration of trips (ie, walking to work or school, to a store or to do an errand, to the bus, or to a neighbor's house) that took at least 10 minutes. Respondents were categorized as walking for transportation (yes) if they reported such walking on ≥5 days in the past week for ≥30 minutes or more each day and as no if they did not.

j Respondents were considered to have engaged in muscle-strengthening activity (eg, lifting weights, calisthenics) (yes) if they reported 2 or more days per week.

**Table 4:** Adjusted^a^ Odds Ratios for Overweight^b^ Among Adults Aged ≥50 Years for Men, Women, and Participants Overall, National Health Interview Survey, 2005

**Characteristic**	Overall, AOR (95% CI)	Men, AOR (95% CI)	Women, AOR (95% CI)
**Smoking status**			
Nonsmoker	1 [Reference]	1 [Reference]	1 [Reference]
Former smoker	1.14 (1.01-1.28)	1.29 (1.07-1.56)	1.08 (0.91-1.27)
Current smoker	0.69 (0.58-0.82)	0.72 (0.55-0.94)	0.72 (0.58-0.91)
**Alcohol intake^c^**			
None	1 [Reference]	1 [Reference]	1 [Reference]
Occasional, light, or moderate	0.99 (0.88-1.12)	1.11 (0.92-1.35)	0.94 (0.80-1.10)
Heavy	0.72 (0.55-0.94)	0.79 (0.52-1.19)	0.73 (0.49-1.09)
**Servings per day of fruits and vegetables^d^**			
<2	1.14 (0.97-1.32)	1.20 (0.97-1.50)	1.04 (0.85-1.27)
2 to <3.5	1.21 (1.06-1.39)	1.27 (1.03-1.56)	1.15 (0.96-1.37)
≥3.5	1 [Reference]	1 [Reference]	1 [Reference]
**Leisure-time physical activity^e^**			
Inactive	1.03 (0.87-1.21)	1.01 (0.79-1.28)	1.04 (0.84-1.30)
Insufficient	1.14 (0.98-1.32)	1.14 (0.90-1.42)	1.16 (0.94-1.44)
Recommended	1 [Reference]	1 [Reference]	1 [Reference]
**Walking for leisure^f^**			
Yes	1 [Reference]	1 [Reference]	1 [Reference]
No	1.12 (0.94-1.32)	0.91 (0.70-1.18)	1.27 (1.01-1.60)
**Walking for transportation^g^**			
Yes	1 [Reference]	1 [Reference]	1 [Reference]
No	1.37 (1.07-1.75)	1.42 (1.02-1.97)	1.36 (0.94-1.98)
**Strength training^h^**			
Yes	1 [Reference]	1 [Reference]	1 [Reference]
No	1.17 (1.01-1.36)	1.02 (0.83-1.27)	1.34 (1.07-1.68)

Abbreviations: AOR, adjusted odds ratio; CI, confidence interval.

a Adjusted for age, sex, race/ethnicity, education level, family income, self-rated health, disability status, and all other variables in the table.

b Refers to participants with a body mass index of 25.0-29.9 kg/m^2^.

c Drinking levels were determined using the *Dietary Guidelines for Americans, 2005* (16), which recommends a sex-specific cutpoint of 1 drink or fewer per day for women and 2 drinks or fewer per day for men. Respondents who consumed alcohol in excess of the recommended levels were considered heavy drinkers.

d Respondents were asked how frequently they consumed the following foods during the past month: fruit, fruit juice, green salad, potatoes (excluding french fries, fried potatoes, and potato chips), and other vegetables. We calculated servings per day of fruits and vegetables and divided results into approximate tertiles.

e Respondents who reported engaging in ≥30 minutes of moderate-intensity physical activity on ≥5 days per week and/or ≥20 minutes of vigorous-intensity physical activity on ≥3 days per week were categorized as meeting the recommended level (17,18). Respondents who reported some activity but at a lower-than-recommended level were categorized as "insufficient," and respondents who reported no physical activity in a usual week were categorized as "inactive."

f Participants were asked whether they walked outdoors for at least 10 minutes at a time for fun, relaxation, or exercise, or to walk a dog.

g Walking for transportation was assessed with questions about respondents' number of days of walking during the previous week and the average total daily duration of trips (ie, walking to work or school, to a store or to do an errand, to the bus, or to a neighbor's house) that took at least 10 minutes. Respondents were categorized as walking for transportation (yes) if they reported such walking on ≥5 days in the past week for ≥30 minutes or more each day and as no if they did not.

h Respondents were considered to have engaged in muscle-strengthening activity (eg, lifting weights, calisthenics) (yes) if they reported 2 or more days per week.

**Table 5:** Adjusted^a^ Odds Ratios for Obesity^b^ Among Adults Aged ≥50 Years for Men, Women, and Participants Overall, National Health Interview Survey, 2005

**Characteristic**	Overall, AOR (95% CI)	Men, AOR (95% CI)	Women, AOR (95% CI)
**Smoking status**			
Nonsmoker	1 [Reference]	1 [Reference]	1 [Reference]
Former smoker	1.17 (1.02-1.33)	1.43 (1.16-1.75)	1.03 (0.86-1.24)
Current smoker	0.46 (0.36-0.57)	0.49 (0.36-0.68)	0.45 (0.35-0.58)
**Alcohol intake^c^**			
None	1 [Reference]	1 [Reference]	1 [Reference]
Occasional, light, or moderate	0.99 (0.85-1.14)	1.28 (1.02-1.61)	0.86 (0.71-1.04)
Heavy	0.55 (0.40-0.76)	0.78 (0.49-1.26)	0.45 (0.28-0.73)
**Servings per day of fruits and vegetables^d^**			
<2	1.23 (1.05-1.45)	1.33 (0.86-2.06)	1.07 (0.86-1.33)
2 to <3.5	1.16 (1.01-1.33)	1.14 (0.75-1.72)	1.00 (0.83-1.19)
≥3.5	1 [Reference]	1 [Reference]	1 [Reference]
**Leisure-time physical activity^e^**			
Inactive	1.30 (1.06-1.60)	1.39 (1.02-1.89)	1.28 (0.99-1.65)
Insufficient	1.35 (1.14-1.61)	1.54 (1.15-2.07)	1.26 (0.99-1.61)
Recommended	1 [Reference]	1 [Reference]	1 [Reference]
**Walking for leisure^f^**			
Yes	1 [Reference]	1 [Reference]	1 [Reference]
No	1.81 (1.46-2.24)	1.42 (1.01-1.99)	2.04 (1.52-2.75)
**Walking for transportation^g^**			
Yes	1 [Reference]	1 [Reference]	1 [Reference]
No	1.47 (1.10-1.96)	1.46 (0.95-2.27)	1.50 (1.02-2.23)
**Strength training^h^**			
Yes	1 [Reference]	1 [Reference]	1 [Reference]
No	1.38 (1.14-1.68)	1.31 (0.96-1.79)	1.36 (1.08-1.72)

Abbreviations: AOR, adjusted odds ratio; CI, confidence interval.

a Adjusted for age, sex, race/ethnicity, education level, family income, self-rated health, disability status, and all other variables in the table.

b Refers to participants with a body mass index ≥30.0 kg/m^2^.

c Drinking levels were determined using the *Dietary Guidelines for Americans, 2005* (16), which recommends a sex-specific cutpoint of 1 drink or fewer per day for women and 2 drinks or fewer per day for men. Respondents who consumed alcohol in excess of the recommended levels were considered heavy drinkers.

d Respondents were asked how frequently they consumed the following foods during the past month: fruit, fruit juice, green salad, potatoes (excluding french fries, fried potatoes, and potato chips), and other vegetables. We calculated servings per day of fruits and vegetables and divided results into approximate tertiles.

e Respondents who reported engaging in ≥30 minutes of moderate-intensity physical activity on ≥5 days per week and/or ≥20 minutes of vigorous-intensity physical activity on ≥3 days per week were categorized as meeting the recommended level (17,18). Respondents who reported some activity but at a lower-than-recommended level were categorized as "insufficient," and respondents who reported no physical activity in a usual week were categorized as "inactive."

f Participants were asked whether they walked outdoors for at least 10 minutes at a time for fun, relaxation, or exercise, or to walk a dog.

g Walking for transportation was assessed with questions about respondents' number of days of walking during the previous week and the average total daily duration of trips (ie, walking to work or school, to a store or to do an errand, to the bus, or to a neighbor's house) that took at least 10 minutes. Respondents were categorized as walking for transportation (yes) if they reported such walking on ≥5 days in the past week for ≥30 minutes or more each day and as no if they did not.

h Respondents were considered to have engaged in muscle-strengthening activity (eg, lifting weights, calisthenics) (yes) if they reported 2 or more days per week.
